# Manifestations of Hydralazine-Induced Vasculitis: A Case Series

**DOI:** 10.7759/cureus.72147

**Published:** 2024-10-22

**Authors:** Christine Sykalo, Riyahd Al-Rubaye, Christopher R Chew, Abdullah Asreb

**Affiliations:** 1 Cardiology, Northeast Georgia Medical Center Gainesville, Gainesville, USA; 2 Internal Medicine, Northeast Georgia Medical Center Gainesville, Gainesville, USA

**Keywords:** anca, case report, drug induced, hydralazine, vasculitis

## Abstract

Hydralazine has been used for decades for the management of hypertension. However, hydralazine has been associated with various side effects, including autoimmune diseases such as hydralazine-induced antineutrophil cytoplasmic antibody-associated vasculitis (ANCA-vasculitis) and lupus-like syndrome characterized by multiorgan involvement and complex clinical manifestations. We present four cases of hydralazine-induced vasculitis (sometimes referred to as drug-induced lupus or DIL) with different presentations, including diffuse alveolar hemorrhage, hemoptysis, and skin rash. Based on variable manifestations, diagnosis can be difficult, and a high index of suspicion is of paramount importance. The final diagnosis is made by kidney biopsy, and treatment includes discontinuation of the offending agent and frequent immunosuppression. We urge caution with the use of hydralazine and choosing alternative antihypertensives when possible.

## Introduction

Hydralazine is a common vasodilator medication that was first introduced on the market in the 1950s. Now, it is used as adjunct therapy in resistant hypertension, heart failure, hypertension in pregnancy, and acute hypertension management in hospitalized patients [1]. Hydralazine is commonly used in the United States, with an increasing number of prescriptions in the last few years, likely due to its affordable cost. The last available data is from 2019, with 6,655,156 annual prescriptions in the US, which increased by 35% from 2017 [2].

Hydralazine-associated antineutrophil cytoplasmic antibodies (ANCA) vasculitis possesses a wide range of clinical features ranging from nonspecific symptoms to several organ involvements, including skin, kidney, and lungs. Early disease recognition and hydralazine discontinuation seem to be the initial approaches to avoid life-threatening complications of vasculitis, such as pulmonary-renal syndrome. Here, we present a case series of four patients diagnosed with hydralazine-induced vasculitis based on different systemic involvement and heterogeneous clinical manifestations.

## Case presentation

Case 1

**Figure 1:**
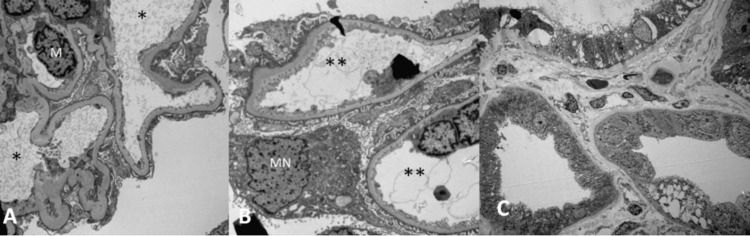
Transmission electron micrograph of kidney biopsy. (A) Cellular glomeruli with mesangial cell (M) and several capillary loops (*). Podocytes display minimal foot effacement and imaging consistent with focal crescentic glomerulonephritis. (B, C) Tubules (**) diffusely exhibit acute injury with luminal ectasia and atrophy. Appreciate fibrinoid necrosis and infiltration of mononuclear cells (MN)

A 34-year-old man with a past medical history of diabetes (DM), hypertension, and chronic kidney disease (CKD) stage 3 presented to the hospital due to shortness of breath for one-week duration. A physical exam revealed diminished breath sounds in the right base, and a chest X-ray showed a right pleural effusion. Home medications included hydralazine 50 mg twice daily, losartan, and allopurinol. He denied smoking or alcohol abuse. The patient was diagnosed with acute or chronic kidney injury due to elevation in serum creatinine from 2.5 mg/dl baseline to 3.5 mg/dL. Urine analysis revealed proteinuria with +1 protein and microscopic hematuria of five red blood cells (RBCs). Pleural fluid analysis showed exudative pleural effusion with a negative fluid culture. Further serologic workup revealed titers positive for ANCA, antibodies directed against myeloperoxidase (anti-MPO), anti-proteinase 3, antinuclear antibodies (ANA), and anti-histone antibodies. The kidney biopsy was postponed because of an elevated partial thromboplastin time (PTT) secondary to the presence of antiphospholipid antibodies (see Table 1 for titers and antibodies). The patient had a diagnosis of possible hydralazine-induced vasculitis with pulmonary manifestation. Hydralazine was ultimately discontinued, and empiric treatment with daily prednisone 60 mg and cyclophosphamide 150 mg daily was started. The renal biopsy had to be performed later, once the PTT normalized. Biopsy results were consistent with the diagnosis of focal crescentic glomerulonephritis and pauci-immune crescentic glomerulonephritis associated (Figure 1). The patient’s serum creatinine returned to his baseline, therapy was tapered, and therapy was discontinued after six months.

**Table 1:** Summary of labs, clinical findings, management, and outcomes CKD: chronic kidney disease; HTN: hypertension; RA: rheumatoid arthritis; lupus: systemic lupus erythematosus; DM: diabetes mellitus; CHF: congestive heart failure; CAD: coronary artery disease; ANA: antinuclear antibody; IF: immunofluorescence; QD: once daily; TID: three times daily

Case	Case 1	Case 2	Case 3	Case 4
Age	34	69	79	63
Gender	Male	Female	Female	Male
Comorbidities	CKD, HTN	RA, Lupus, DM	CKD, HTN, DM, CHF, CAD	DM, HTN, CHF, CKD
Highest hydralazine dose	50 mg	100 mg TID	100 mg TID	75 mg TID
Duration of hydralazine therapy	Unknown	>4 years	>7 years	>5 years
Clinical features	Dyspnea with pleural effusion	Productive cough, fever, dyspnea on exertion	Hemoptysis and shortness of breath	Shortness of breath, dyspnea on exertion, and orthopnea
BUN, mg/dL Reference range: 5.0-23.0 mg/dL	41	63	102	94
Serum Cr, mg/dL Reference range: 0.80-1.30 mg/dL	3.5	1.51	3.10	4.84
Baseline Cr, mg/dL	2.15	1.0	1.80	1.50-1.80
Urinalysis RBC Reference range: 0-5 RBC	5	6	10	120
Urinalysis protein Reference range: negative	1+	1+	2+	2+
Protein creatinine ratio Reference range < 0.2 mg/mg	202	1.18	1.2	0.47
MPO titer, AU/mL Reference range < 19 negative, 20-25 equivocal, >26 positive	162	169	71	150
PR3 titer, AU/mL Reference range < 19 negative, 20-25 equivocal, >26 positive	45	2	20	435
C3 (normal: 85-170 mg/dL)	135	109	71	128
C4 (normal: 16-40 mg/dL)	27.4	22.4	2.65	13.7
ANA titer/IF pattern	Negative	Negative	1:320 (Diffused)	1:320 (homogenous)
Lupus anti-coagulant/anticardiolipin	Not done	Positive	Not done	Not done
Anti-dsDNA	Not done	Positive	Negative	Positive
Anti-histone	Positive	Negative	Positive	Positive
Biopsy day	Day 30	Day 12	Day 20	Day 6
Biopsy findings	Focal crescentic glomerulonephritis, pauci-immune type	Acute tubular injury, arteriosclerosis	Focal segmental crescentic glomerulonephritis, pauci-immune type	Focal necrotizing and sclerosing glomerulonephritis, acute tubular injury
Treatment	Prednisone 60 mg QD and cyclophosphamide 150 mg QD	Solumedrol 60 mg Q6 hours and rituximab for 4 doses. Discharged on prednisone 20 mg PO BID	Rituximab 650 mg dose and methylprednisolone 1 g x 3 days, then prednisone 60 mg QD	Initiation therapy with 2 doses of rituximab 1 gm 14 days apart. Received Solumedrol 500 mg x 3 days, followed by prednisone 60 mg QD.
Hemodialysis	No	No	No	Once
Length of stay	5 days	24 days	22 days	12 days
Outcome	Discharged	Discharged	Passed away	Discharged
Creatinine 2 months post admission	2.6	Deceased	Deceased	2.34
Hydralazine stopped on what day of admission	Day 3	Day 12	Day 18	Day 4

Case 2

**Figure 2:**
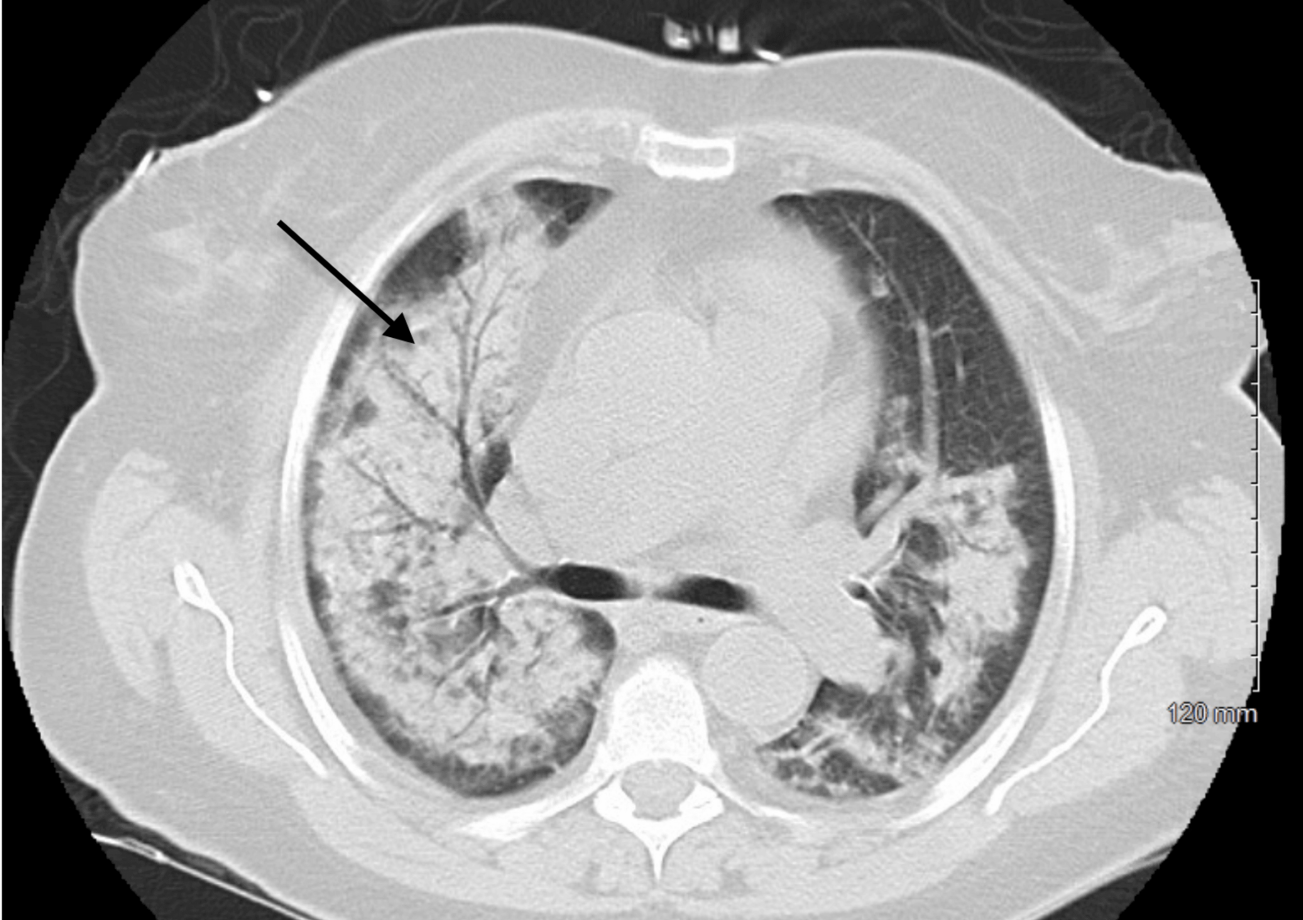
Extensive right greater than left confluent airspace opacities with air bronchograms and subpleural sparing, as demonstrated by the arrow. This is compatible with multifocal pneumonia in the appropriate clinical scenario. ARDS and edema are also considered ARDS: acute respiratory distress syndrome

A 69-year-old woman with a history of CKD stage II, fibromyalgia, DM, hypertension, systemic lupus erythematosus, and rheumatoid arthritis presented to the hospital due to productive cough, fever, and dyspnea on exertion of four days duration. The patient had no history of tobacco or alcohol use. Home medications were notable for spironolactone, minoxidil, hydrochlorothiazide, hydralazine, clonidine patch, metformin, pravastatin, and levothyroxine. Chest X-ray showed multifocal patchy opacities bilaterally. The coronavirus disease 2019 (COVID-19) and viral respiratory panel were negative. Her hypoxia worsened despite treatment for community-acquired pneumonia. Further workup, including urine studies, showed moderate blood, 1+ protein, six RBCs, and a urine protein creatinine ratio of 1.1 g/g of creatinine, while serum creatinine was normal at 1.01 mg/dL. Hemoglobin dropped to 6.6 g/dL during the early hospital stay. A chest computed tomography (CT) showed diffuse alveolar hemorrhage (DAH) versus multifocal pneumonia (Figure 2). Serologies were positive for ANCA, myeloperoxidase (MPO), immunoglobulin G (IgG), anti-double-stranded deoxyribonucleic acid (DS-DNA) IgG, and lupus anticoagulant panel. Due to suspicion of pulmonary renal syndrome (PRS), a renal biopsy was performed and showed features of acute tubular necrosis. The etiology of her presentation was presumed to be secondary to long-term hydralazine. The patient was diagnosed with hydralazine-induced P-ANCA pulmonary vasculitis with suspected DAH. She was treated with high-dose systemic steroids and rituximab infusions while hospitalized. The patient's clinical condition improved significantly during the 24-day hospitalization, and she was discharged with 4 L supplemental oxygen.

Case 3

A 79-year-old woman with a history of congestive heart failure (CHF) with preserved ejection fraction, coronary artery disease, hypertension, stage III CKD, and DM presented to the hospital due to hemoptysis and shortness of breath that started one day prior to presentation. The patient had no history of alcohol or tobacco use, and home medications were notable for metoprolol succinate, amlodipine, insulin, hydralazine, and apixaban. She was breathing normally on room air. The patient was noted to have anemia of chronic disease and elevated creatinine, which was at a baseline of 2.26 mg/dL. The echocardiogram was unremarkable; however, a ventilation-perfusion scan showed evidence of bilateral pulmonary emboli. She was started on a heparin infusion.

**Figure 3:**
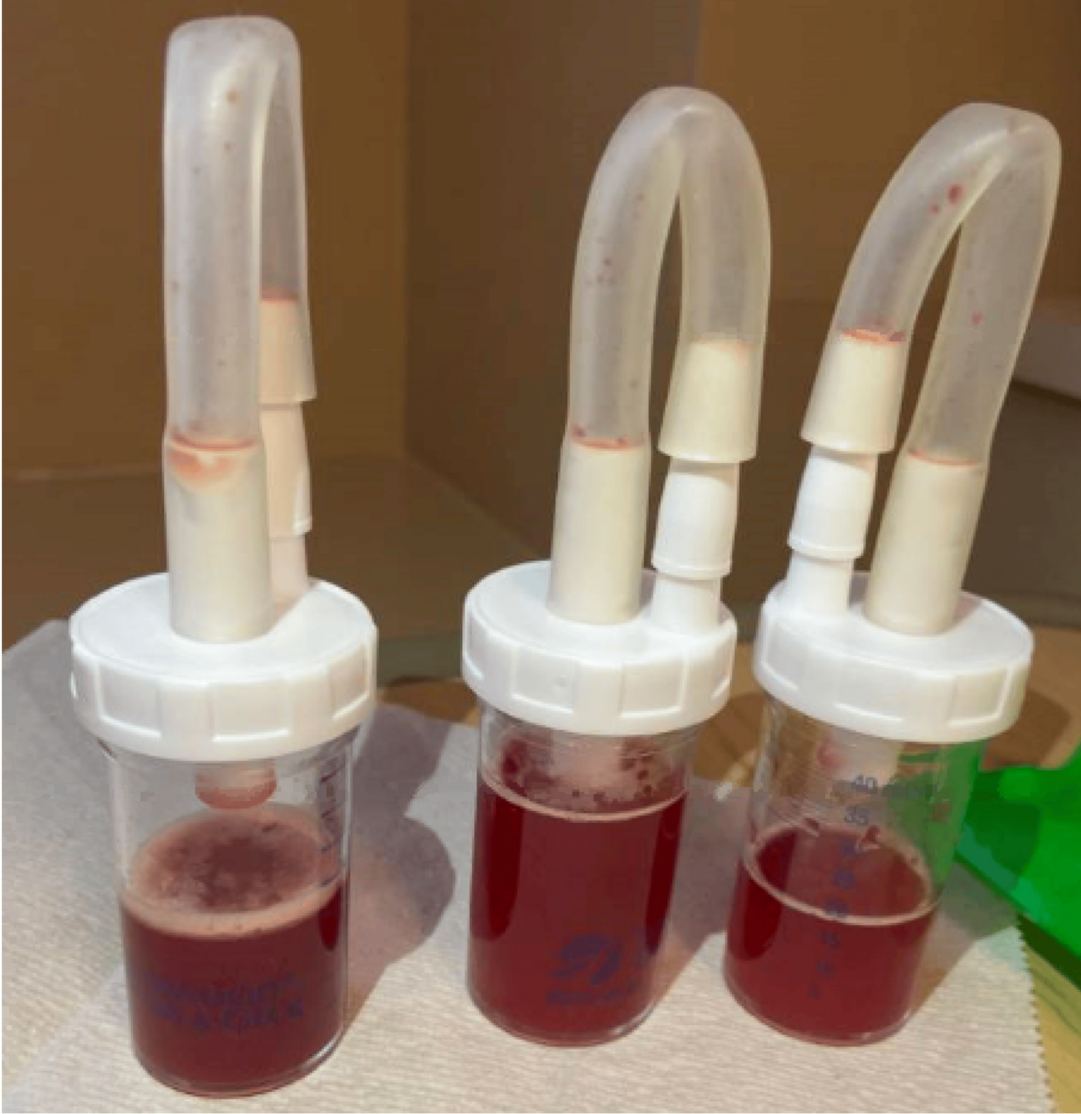
Bronchoscopy specimen consistent with diffuse alveolar hemorrhage

She had worsening respiratory status, and bronchoscopy revealed evidence of DAH with bloody bronchoalveolar lavage (Figure 3). The patient developed acute or chronic kidney injury that worsened despite aggressive hydration. Her urinalysis showed clear urine with moderate blood and 2+ protein. Laboratory studies were positive for ANCA-MPO. The kidney biopsy results revealed focal segmental crescentic and necrotizing glomerulonephritis, suggesting pauci-immune ANCA-associated glomerulonephritis. She had been on hydralazine for years, and a diagnosis of hydralazine-induced ANCA vasculitis was made. The patient was started on weekly rituximab and high-dose steroids. The patient’s overall condition was improving; unfortunately, the patient suffered respiratory arrest secondary to mucus plugging and subsequently passed away.

Case 4

**Figure 4:**
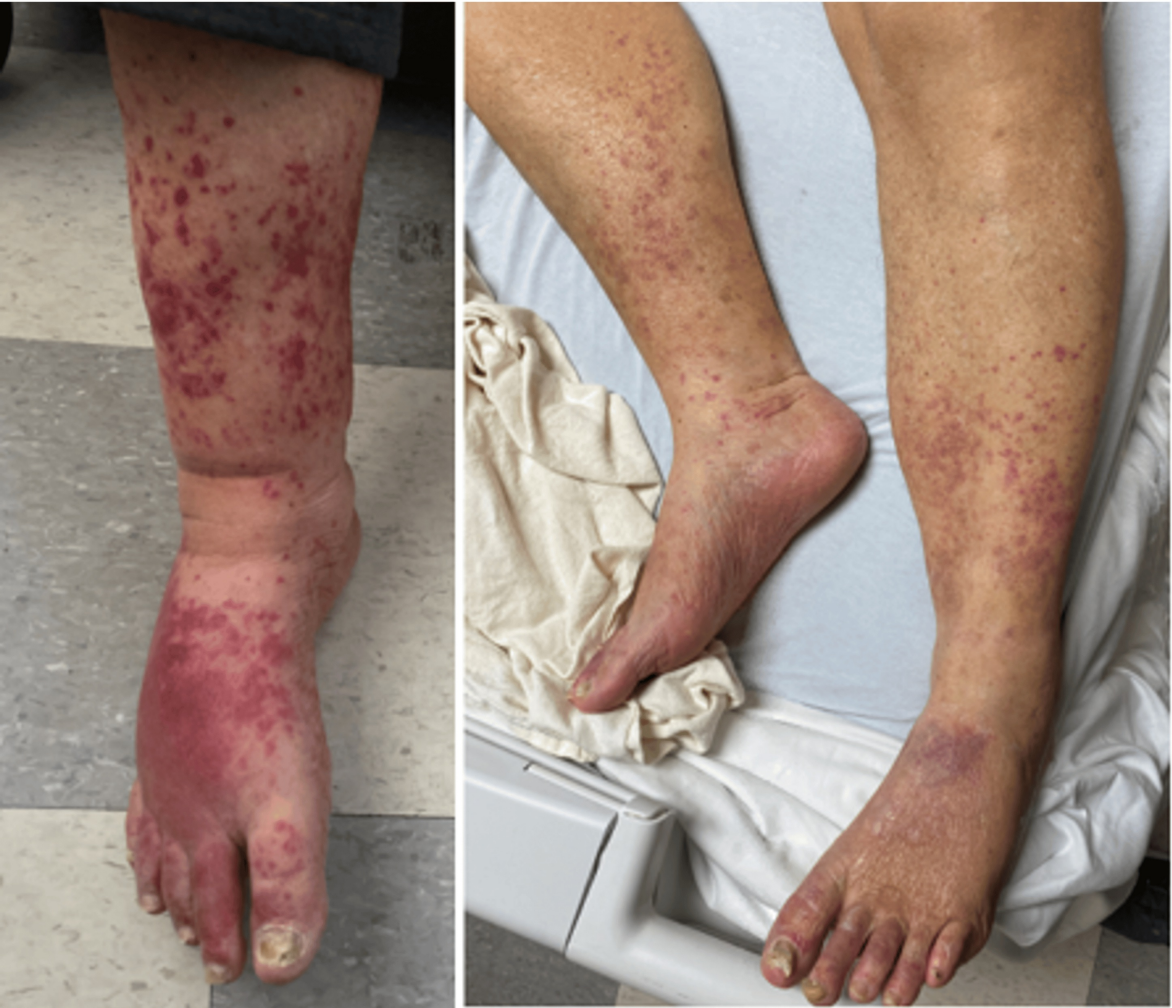
Vasculitis day prior to starting steroids followed by day 2 of receiving high dose steroids

A 63-year-old man has a medical history of CHF, stage 3 CKD, hypertension, DM, and prior ischemic stroke and presented with complaints of shortness of breath. The patient reported no history of smoking or alcohol use. Home medications included furosemide, hydralazine, clonidine, losartan, amlodipine, and atorvastatin. The patient had recent hospital admission due to acute heart failure exacerbation with fluid overload. On this admission, he was diagnosed with acute hypoxemia, respiratory failure, pleural effusions, and acute kidney injury. He had elevated serum creatinine at 4.84 mg/dL on admission, while baseline creatine was noted to be 1.5 mg/dL. Initially, the patient was diagnosed with acute heart failure exacerbation and acute severe cardiorenal syndrome with moderate uremic pericardial effusion that required one session of hemodialysis. On day three of the hospital admission, the patient developed a red macular, non-blanchable, non-pruritic rash on both lower extremities. His urine analysis showed 2+ protein and 120 RBC/hpf, with hyaline and granular casts. The urine protein creatinine ratio was 0.47, and the serological workup was positive for anti-double-stranded DNA, ANCA MPO/PR3, and anti-histone. Hydralazine-induced vasculitis was suspected, necessitating a kidney biopsy, which showed necrotizing vasculitis. The patient was immediately started on steroids, with significant improvement in the rash by day two (Figure 4). In the setting of ANCA-associated vasculitis, the patient was started on rituximab with high-dose steroids. The patient improved without requiring further hemodialysis and was discharged with close outpatient follow-up.

Our report presents four Caucasian patients, two male and two female. All the patients had been on doses of at least 50 mg of hydralazine or higher. All had hydralazine exposure for more than four years except one; his duration of exposure was unknown. Two patients were diagnosed with DAH; one had non-resolving pulmonary edema, and the last had a pleural effusion. All titers and labs are summarized in Table 1. The average length of stay was 16 days, and biopsies were done between days 6 and 30 of diagnosis recognition. Three of the patients had focal crescentic glomerulonephritis and one had acute tubular necrosis. All patients received steroids; three received rituximab, and one received cyclophosphamide. Three patients were discharged, and one passed away on that admission. At the two-month mark post-discharge, only two out of four patients were living. The two living patients had new baseline creatinine levels that increased from the previous.

## Discussion

Hydralazine is a rare cause of vasculitis with an incidence of 10-20 cases per million [3]. Hydralazine-induced anti-neutrophilic cytoplasmic antibody (ANCA) vasculitis is dose-dependent and has an incidence of 5.4% in patients with 100 mg/day to 10.4% with 200 mg/day for more than three years [4]. Drug-induced vasculitis (DIV) usually presents with nonspecific symptoms, including fatigue, weakness, and weight loss. Patients can present with rapidly progressive crescentic glomerulonephritis, cutaneous vasculitis, and various types of pulmonary disease [5].

The difficulty of identifying DIV is the variety and overlap of symptoms. Since incidence remains low [6], there aren’t many described cases of hydralazine-induced vasculitis. A publication by Yokogawa and Vivino in 2009 reviewed 68 cases of hydralazine-induced vasculitis in the medical literature with a mean age of 64 years old. In their reviewed cases, all patients had antibodies to MPO, and 44% had low complement counts. They noted that 81% of patients had kidney involvement, 19% had lung involvement, and 25% had cutaneous vasculitis [5]. In the 25% of patients who present with skin manifestations, DIV may be recognized more readily. The most common skin manifestation is palpable purpura; however, urticaria, skin ulceration and necrotic plaques, livedo reticularis, and skin nodules can also be seen [7]. On the other hand, patients without skin manifestations require a high clinical index of suspicion. Overall, the diagnosis relies on positive serologies and ultimately renal histopathology.

While skin findings are infrequently present, the most common manifestation of hydralazine-induced vasculitis is hydralazine-associated glomerulonephritis [5]. Most of these patients develop crescentic necrotizing nephritis [6,8]. Unfortunately, crescentic glomerulonephritis is the most severe form of glomerular inflammation with histopathological findings of proliferating parietal epithelial cells and infiltrating leukocytes, extra-capillary cells, and plasma proteins within Bowman’s space with inflammation and necrosis [9].

Different forms of pulmonary disease have been reported in the literature since 1992 and include bilateral interstitial edema, pleural effusion, and PRS [8]. PRS is defined as the presence of pauci immune glomerulonephritis with DAH. PRS is usually caused by an autoimmune process; however, it was first attributed to hydralazine and described by Yokogawa and Vivino in 2009 [5].

The possible risk factors for DIV include female sex, history of thyroid disease, and longstanding history of hydralazine use [10]. Both autoimmune conditions and increased dosing lead to drug accumulation, especially in slow acetylator individuals [10]. Hydralazine inhibits DNA methylation, and this hypomethylation process interferes with the PR3 and MPO silencing processes. The result is an expression of the autoantigens in neutrophils [4,10-11]. Anti-MPO (P-ANCA) plays a key role in all cases of hydralazine-induced PRS, and most of the cases have high titers. In contrast, PR3 has variable titers, either low or high. Other serologies include positive anti-histone antibodies and positive anti-DS-DNA [6].

Hydralazine-associated glomerulonephritis is the most common manifestation of hydralazine-induced vasculitis [5], and the majority of patients display crescentic necrotizing nephritis [6]. Diagnosis of hydralazine-associated glomerulonephritis is challenging due to nonspecific symptoms, such as fatigue, weakness, and decreased appetite. These symptoms often resemble more common inpatient diseases, such as diabetic nephropathy, CHF exacerbation, cardiorenal syndrome, and anemia. Due to the broad presentations of vasculitis, a high index of suspicion must be maintained during the workup of patients with pulmonary issues in association with an acute kidney injury. Patients must also be educated on the possible detrimental effects of hydralazine use [9]. 

Previously, there have been 10 reported cases of misdiagnosed ANCA-induced glomerulonephritis that were initially labeled as diabetic nephropathy [12]. Three of our patients had a history of diabetic nephropathy with CKD and were later diagnosed with ANCA-glomerulonephritis as well. Worsening CKD tends to be the initial diagnosis in several cases of DIV because most of these patients already have CKD and the presentation is non-specific. Additionally, severe anemia can lead to unnecessary tests and malignancy screening, which may further delay proper diagnosis [13].

Pulmonary manifestation in DIV or PRS tends to be diagnosed first as CHF exacerbation or pleural effusion because many patients present with shortness of breath and leg edema [1,14]. Our report showed two patients who were initially treated for CHF exacerbation. A proper diagnosis was later obtained due to worsening renal function, leading to kidney biopsies.

Prior publications have also shown that many patients suffered from worsening renal failure, which subsequently led to kidney biopsy and the establishment of the correct diagnosis [14]. However, the time between admission and kidney biopsy was not commonly reported in the literature [15]. One case reported a kidney biopsy being performed on day 3, because the patient had severe AKI with anuria, following mechanical intubation and bronchoscopy. In that specific case, the patient was discharged by day 14 [14]. Our patients’ admission times were variable from 5 days to 24-day stays.

Treatment of DIV includes cessation of hydralazine and early initiation of immunosuppressant therapy. Many documented cases show patients placed on dialysis indefinitely. In severe cases like this, aggressive management with immunosuppressive regimens such as corticosteroids, rituximab, or cyclophosphamide is needed [1,6].

## Conclusions

There are many factors posing a diagnostic challenge in DIV. Notably, failure to perform medication reconciliations during admission may lead to delay of care and diagnosis and is laborious, as patients are often unsure of what medications they take at home. Additionally, the presence of extensive overlap of common symptoms triggers bias and leads to patients being treated for presumed acute heart failure exacerbation or worsening CKD. This can lead to a delay in stopping the hydralazine and worsen the patient's condition. The cardiorenal syndrome can further delay the diagnosis unless the patient develops PRS and skin manifestations of vasculitis, which seems to be the only features that truly facilitate a diagnosis without evidence of nephrotic or nephritic syndromes. With common things being common, DIV is often not on the initial diagnosis of clinicians whose patients have an AKI. While the clinician orders routine workup for AKI, the offending agent is often continued, leading to a delay in proper treatment. This series is meant to demonstrate the wide variety of symptoms and complaints that patients may present with when having a pulmonary-renal pathology. In conclusion, caution is recommended with medications known to induce vasculitis, specifically hydralazine, as it is dose-dependent.

## References

[1] Aeddula NR, Pathireddy S, Ansari A, Juran PJ (2018). Hydralazine-associated antineutrophil cytoplasmic antibody vasculitis with pulmonary-renal syndrome. BMJ Case Rep.

[2] Kane Kane, S. P. (2022). Hydralazine - drug usage statistics, Clincalc Drugstats database. https://clincalc.com/DrugStats/Drugs/Hydralazine.

[3] Ntatsaki E, Watts RA, Scott DG (2010). Epidemiology of ANCA-associated vasculitis. Rheum Dis Clin North Am.

[4] Pendergraft WF 3rd, Niles JL (2014). Trojan horses: drug culprits associated with antineutrophil cytoplasmic autoantibody (ANCA) vasculitis. Curr Opin Rheumatol.

[5] Yokogawa N, Vivino FB (2009). Hydralazine-induced autoimmune disease: comparison to idiopathic lupus and ANCA-positive vasculitis. Mod Rheumatol.

[6] Timlin H, Liebowitz JE, Jaggi K, Geetha D (2018). Outcomes of hydralazine induced renal vasculitis. Eur J Rheumatol.

[7] Fiorentino DF (2003). Cutaneous vasculitis. J Am Acad Dermatol.

[8] Lee RW, D'Cruz DP (2010). Pulmonary renal vasculitis syndromes. Autoimmun Rev.

[9] Radić M, Martinović Kaliterna D, Radić J (2012). Drug-induced vasculitis: a clinical and pathological review. Neth J Med.

[10] Cameron HA, Ramsay LE (1984). The lupus syndrome induced by hydralazine: a common complication with low dose treatment. Br Med J (Clin Res Ed).

[11] Deng C, Lu Q, Zhang Z, Rao T, Attwood J, Yung R, Richardson B (2003). Hydralazine may induce autoimmunity by inhibiting extracellular signal-regulated kinase pathway signaling. Arthritis Rheum.

[12] Jennette JC, Falk RJ (2014). Pathogenesis of antineutrophil cytoplasmic autoantibody-mediated disease. Nat Rev Rheumatol.

[13] Agarwal G, Sultan G, Werner SL, Hura C (2014). Hydralazine induces myeloperoxidase and proteinase 3 anti-neutrophil cytoplasmic antibody vasculitis and leads to pulmonary renal syndrome. Case Rep Nephrol.

[14] Al-Abdouh A, Siyal AM, Seid H, Bekele A, Garcia P (2020). Hydralazine-induced antineutrophil cytoplasmic antibody-associated vasculitis with pulmonary-renal syndrome: a case report. J Med Case Rep.

[15] Paley MA, Edrees F, Kudose S, Gaut JP, Ranganathan P, Vijayan A (2019). Successful use of rituximab for hydralazine-induced anti-neutrophil cytoplasmic antibodies-associated vasculitis. Saudi J Kidney Dis Transpl.

